# Next Generation Wireless Technologies for Internet of Things

**DOI:** 10.3390/s18010221

**Published:** 2018-01-14

**Authors:** Giovanni Pau, Claude Chaudet, Dixian Zhao, Mario Collotta

**Affiliations:** 1 Faculty of Engineering and Architecture, Kore University of Enna, Cittadella Universitaria, 94100 Enna, Italy; mario.collotta@unikore.it; 2 Department of Computer Science and Mathematics, Webster University Geneva, 15, Route de Collex, 1293 Bellevue, Switzerland; chaudet@webster.ch; 3 School of Information Science and Engineering, Southeast University (SEU), Nanjing 211189, China; dixian.zhao@seu.edu.cn

## 1. Introduction

In the fast-growing Internet of Things (IoT), applications from personal devices to industrial instruments and sensors connect wirelessly to the Internet. Considering that a broad diversity of use cases in different environments with specific requirements can be developed, it is clear that no single wireless standard can abundantly predominate. With many standards available on the market, spread over various frequency bands and employing diverse communication protocols, the choice of the best wireless connectivity technology for an IoT application can be quite challenging.

The first issues to be evaluated are the frequency bands and global regulations. In fact, in most regions, the available spectrum assignment is subdued to a licensing scheme, which implies that users have to obtain a license from the local regulator to transmit signals in a selected frequency channel. On the contrary, several frequency bands have been designated for Industrial, Scientific, and Medical (ISM) applications. These bands are unlicensed and differ slightly from country to country. In recent years, the popular ISM bands have involved 433 MHz, 868 MHz, 915 MHz, 2.4 GHz, and 5 GHz. Nevertheless, as a standard rule, it is well-known that higher-frequency bands offer a broader bandwidth and hence make broader bandwidth and more channels available. As a consequence, they can serve more extended networks and handle a higher data throughput. Contrariwise, lower-frequency radio waves propagate better than higher-frequency ones and can thus achieve a longer range, especially inside buildings.

Another characteristic to be analyzed is the communication protocol. In fact, for several years, numerous protocols have been developed following a layered network implementation. This choice, on the one hand, includes complexity and needs more code and memory. Furthermore, it also implies data overhead because each layer expects further framing and control messages. Nonetheless, on the other hand, layered networks facilitate more flexibility and scale. For instance, it is useful to note that a simple network scheme, with no or limited layering, is represented by a proprietary application protocol, operating directly over the physical layer equipped with a simple radio transceiver. It is clear that this type of solution can be pretty efficient, but it can be used only in simple, single-function networks.

The network range is undoubtedly another feature to take into account. A network’s range is commonly categorized in 4 categories: Personal Area Networks (PANs), Local Area Networks (LANs), Neighborhood Area Networks (NANs), and Wide Area Networks (WANs). PANs usually are wireless and cover a range of about 10 m. A direct consequence of these features is that wireless PAN devices typically have low radio transmission power and operate on tiny batteries. On the contrary, LANs can be wired or wireless and a combination of the two technologies is also possible. It is useful to note that wireless LANs (WLANs) ordinarily cover a range up to 100 m. In most cases, NANs are wireless and can reach more than 25 km. In this case, they transmit with high power levels but usually carry low data traffic. Lastly, WANs are disseminated across an extensive area. As it is known, the Internet is considered a WAN and involves a heterogeneous mix of wired and wireless connections.

The way in which wireless nodes in a network are organized and how they connect to each other, i.e., the topology, is another peculiarity to evaluate. The typical network topologies are star and mesh. In a star-based network, all nodes are connected to a central one, which typically serve as the gateway to a wider network. On the contrary, in a mesh network, every node can connect to various other ones. In this case, one or more nodes can work as a gateway. The main advantage of a mesh network is that it can increase the range through many hops while keeping a low radio transmission power. Moreover, in a mesh topology, it is also feasible to obtain a higher reliability by providing more than one path to deliver data through the network. Nevertheless, in a mesh network, it is necessary to note that routing nodes usually need extra memory and processing power to establish the routing functions. A direct consequence of these requirements is that mesh networks are likewise complicated and expensive to plan. Another drawback is that, compared to a star topology, mesh networks can exhibit longer delays when routing data from a remote node through the mesh. The network size, i.e., the maximum number of connected nodes, represents also a significant problem in system design. Several technologies support up to 20 connections, while others can carry thousands of connections.

Finally, the fundamental challenge in communication systems is represented by the interoperability, i.e., the capacity of devices from different vendors to interact each other. Facing this difficulty is the primary goal of the several standards organizations that establish specifications and testing methods. In fact, some standards specify one or several network layers, while others describe the whole end-to-end network specifications.

The goal of this Special Issue has been to bring together a broad range of methods and technical fields to resolve open problems and hurdles typical applications of wireless technologies for the IoT, efficiently combining novel solutions, and converging on performance evaluations and comparisons with existing standards. In the next section, we present a brief review of the papers published, approximately organizing them according to thematic areas.

## 2. A Review of the Contributions in This Special Issue

In the last years, various research efforts have been carried out to merge the IoT with smart city environments. The goal to shape a city “smart” is rising as a potential solution to decrease the issues caused by the urban population growth and fast urbanization. Information and Communications Technologies (ICT) can unquestionably be an outstanding assistance in developing smart cities. In fact, some papers published in the special issue focused on these research fields.

The authors of [1] present a novel solution able to sustain the development and dynamic deployment of infrastructure-less wireless applications in sparse smart city environments. The solution introduced by the authors enables the dynamic control of mobile nodes by creating new single-hop links and dispatching packets at multi-hop distance. Moreover, it also lets software components on mobile nodes to be deployed and upgraded on-the-fly. The results obtained through a working implementation of the proposed middleware are promising to the point that the authors, in their future works, will focus on on the containerization of the proposed solution as well as of specific sensor/controller components.

A novel methodology to distinguish the electromagnetic field induced by wireless networks is presented in [2]. The authors exploit the functionalities of a smart city platform to use some low-complexity probes. The analysis of the information gathered during a whole year validates the proposed technique regarding the characterization of large areas, close-by dosimeters, and the exposure induced by the different wireless communication technologies. In the scenario of Vehicular Ad-Hoc Networks (VANETs), the authors of [3] present an underlay Radio Resource Management approach that can enhance cellular spectral capability, with a minimum influence on cellular communications, and, at the same time, guarantees the different Quality of Service (QoS) requirements of Intelligent Transportation Systems (ITS) applications. Simulations results, highlight the benefits of the spectral efficiency provided by the presented approach. Moreover, the improvement of energy efficiency is the goal of the authors in their future works.

Other authors have concentrated their research works on proposing specific improvements concerning the protocol structure or hardware components. For instance, the potential of the Sub-GHz wireless communications for future IoT wearable applications is examined in [4]. The authors focus on the antenna gain limitation and, for this reason, several tests are conducted to compare two bands. Results highlight that the 915 MHz band offers a comparable link budget when compared to the 2.45 GHz one. Further evaluation is carried out among different wireless protocols and radio transceivers, and the results reveal that average current consumption is an active function of sleep current and sampling rate. The authors also present an antenna design developed to work both in free-space and on-human wrist scenarios.

An authentication and key management mechanism for the establishment of a security association between a resource-constrained IoT device and an access point is introduced in [5]. The proposed mechanism can reduce the number of cryptographic processes needed on the IoT device by transferring the mutual authentication and the key derivation functions processes of the device to the station-side authentication server. Results show that the mechanism presented by the authors decreases almost all the computational costs, the cryptographic functions can be replaced with new ones without affecting the resource-constrained devices, and a better scalability of the access point even with a large number of stations can be achieved.

In [6], the primary aim of the authors is to prove that multi-model distribution of the received signal strength indicator (RSSI) is linked to the signals’ modulation schemes and medium access mechanisms, and RSSI from different technologies may present very peculiar characteristics. An experimental study is carried out, and the results prove that even RSSI obtained at a sub-Nyquist sampling rate can give sufficient peculiarities to separate technologies such as Wi-Fi, Long Term Evolution (LTE), Digital Video Broadcasting-Terrestrial (DVB-T) and Bluetooth. On the contrary, the authors of [7] present a state-of-the-art survey explicitly intended for examining middlewares with the quality of context support. Furthermore, they propose a middleware of this type, suitable for IoT applications, which combines the use of a mobile gateway with a data distribution service. The main aim of the suggested middleware is to meet several requirements related to the specific IoT application. The performance evaluation shows that the data processing time of the middleware components is relatively low. Also, a small impact on the total delivery time is needed for communication between data producers and consumers.

The most significant number of papers accepted in the special issue has concentrated on proposing innovative applications, in different contexts, suitable for the Internet of Things. For instance, the authors of [8] suggest the use of virtual objects to control and manage the various resource-constrained objects that define the IoT, with the aim to determine those that can accomplish some specific tasks guaranteeing the required quality of information. To this end, they propose a consensus-based algorithm where the resources of these objects are designated for the execution of tasks so that the workload is allocated in a fair way. Tests on real devices prove that the proposed algorithm can obtain a concrete improvement concerning the average lifetime.

A Smart Collaborative Caching (SCC) scheme is introduced in [9] with the aim to support an information-oriented IoT establishment. The authors provide details on several features, such as the cluster composition, content processing, and position management. Moreover, four representative SCC actions and appropriate algorithms are also analyzed. The results obtained in a validation topology composed of two scenarios prove that the performance of the proposed scheme, concerning total packet number and average transmission latency, can outperform that of the original ones. As future works, the authors aim to examine the applicability of SCC in numerous scenarios and further optimize the data exchange processes.

The authors of [10] present the results from three-years of constant monitoring of environmental conditions employing a wireless sensor network established in an art museum. The proposed solution is based on several sensors to gather the temperature and air flow and to evaluate microclimate changes using physics-based and statistical models. The results exhibited by the authors confirm not only the effectiveness of monitoring through their platform but that in specific contexts the installation of a wireless sensor network may be the best choice since it is less invasive compared to a wired solution. On the contrary, the problem of estimating the location of multiple individuals moving and interacting in an indoor space is analyzed in [11]. In this case, the authors propose a multi-sensor data-fusion framework, relying on a unifying location-estimate representation as a confidence map of the indoor space. The effectiveness concerning the deployment of a wireless sensor network is also proven in this context because the results regarding the indoor localization are very encouraging.

The authors of [12] focus on the problem of target location in belt-type sensor networks. In the proposed solution, an energy-efficient strategy is utilized to dispose of IoT nodes. Moreover, a decision mechanism of the broadcasting data is employed to enhance the security and reliability of the positioning information. The obtained results reveal that the proposed algorithm efficiently improves the speed and precision of the positioning, also reducing the possibility of the anchor nodes suffering abnormal interference, and meeting the needs of energy efficiency. Finally, a monitoring system, based on a line information reader and line information generator for optical fiber communication networks, is proposed in [13]. The proposed setup applies to control and manage defaulted optical lines. Simulation results demonstrate that the smaller the radius of curvature and the longer the wavelength of the transmission optical signal, the more the optical radiation field leakage of the fiber bending.

## 3. Conclusions

This Special Issue has pointed out that the application of wireless technologies for the Internet of Things charm much attention in several research fields. In fact, nowadays, there are many wireless technologies available in the world. It is clear that each one has advantages, and, at the same time, none is flawless. The question on which technology is the best for most applications remains open. In fact, every application scenario involves specific requirements concerning the range, power consumption, throughput, and network topology. Besides, further considerations include the cost, ease of integration, and security. Several papers have been submitted in this Special Issue to cope with many of these problems, presenting new solutions and innovative methods. The accepted papers have proposed smart solutions, intelligent algorithms and original network models that produce a substantial augmentation to the literature that deals with the application of wireless technologies for the Internet of Things.
